# Prospects and Potential for Chimerism Analysis after Allogeneic Hematopoietic Stem Cell Transplantation

**DOI:** 10.3390/cells13110993

**Published:** 2024-06-06

**Authors:** Saori Miura, Koki Ueda, Keiji Minakawa, Kenneth E. Nollet, Kazuhiko Ikeda

**Affiliations:** 1 Department of Clinical Laboratory Sciences, Fukushima Medical University School of Health Sciences, Fukushima 960-8516, Japan; 2 Department of Blood Transfusion and Transplantation Immunology, Fukushima Medical University School of Medicine, Fukushima 960-1295, Japan

**Keywords:** chimerism analysis, hematopoietic stem cell transplantation, mixed chimerism

## Abstract

Chimerism analysis after allogeneic hematopoietic stem cell transplantation serves to confirm engraftment, indicate relapse of hematologic malignancy, and attribute graft failure to either immune rejection or poor graft function. Short tandem repeat PCR (STR-PCR) is the prevailing method, followed by quantitative real-time PCR (qPCR), with detection limits of 1–5% and 0.1%, respectively. Chimerism assays using digital PCR or next-generation sequencing, both of which are more sensitive than STR-PCR, are increasingly used. Stable mixed chimerism is usually not associated with poor outcomes in non-malignant diseases, but recipient chimerism may foretell relapse of hematologic malignancies, so higher detection sensitivity may be beneficial in such cases. Thus, the need for and the type of intervention, e.g., immunosuppression regimen, donor lymphocyte infusion, and/or salvage second transplantation, should be guided by donor chimerism in the context of the feature and/or residual malignant cells of the disease to be treated.

## 1. Introduction

Allogeneic hematopoietic stem cell transplantation (allo-HSCT), including bone marrow transplantation (BMT), peripheral blood stem cell transplantation (PBSCT), and cord blood transplantation (CBT), can cure various non-malignant and malignant hematologic disorders by replacing patient hematopoietic cells with donor-derived normal hematopoietic cells. Although allo-HSCT safety has improved through decades of practice, comorbidities and mortality risks persist due to relapse of the underlying malignancy, infection, graft-versus-host disease (GVHD), and graft failure [1]. 

**Figure 1:**
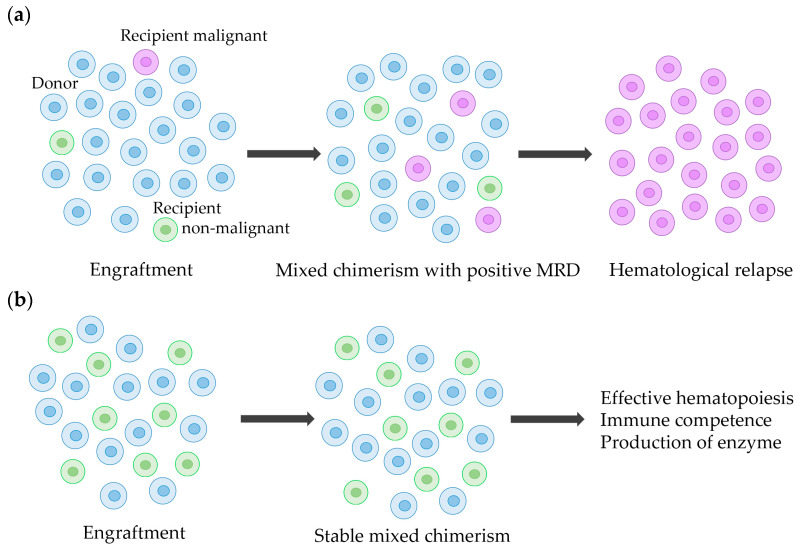
Schema of donor and recipient cells after allogeneic hematopoietic stem cell transplantation (allo-HSCT). (**a**) Relapse of AML from recipient-derived malignant cells after allo-HSCT. At the time of engraftment, the proportions of recipient-derived non-malignant and malignant cells are lower than detectable levels by recipient chimerism or minimal residual disease (MRD) tests (left panel). After molecular evidence of relapse with mixed chimerism and detectable MRD (middle panel), recipient-derived malignant cells proliferate to hematological relapse (right panel). (**b**) Stable mixed chimerism in non-malignant disease after allo-HSCT. Non-malignant recipient-derived cells do not interfere with the functional roles of donor-derived cells, such as hematopoiesis, immune competence, and enzyme production after engraftment of allo-HSCT.

Chimerism analysis, which assesses the proportions of hematopoietic cells derived from the donor (donor chimerism) and recipient (recipient chimerism), is crucial for successful allo-HSCT [2,3]. Complete donor chimerism presumes the absence of hematopoietic cells of recipient origin, whereas mixed chimerism is characterized by the existence of both donor and recipient hematopoietic cells [3]. Mixed chimerism is associated with graft rejection and relapse of malignant disease due to the persistence of recipient-derived cells, but mixed chimerism results vary among different methods of chimerism analysis. Moreover, the clinical impact of mixed chimerism depends on the underlying disease and clinical status. For instance, relapse of acute myeloid leukemia (AML) can arise from a small number of recipient-derived cells carrying the disease-driver mutation, whereas stable mixed chimerism can allow effective hematopoiesis with transfusion independence in non-malignant inherited hemaglobinpathies when donor-derived progenitor cells produce a sufficient number of erythrocytes (Figure 1). Pre-emptive donor lymphocyte infusion (DLI) may be a therapeutic option for patients with mixed chimerism to prevent relapse of high-risk AML [4,5,6]. In contrast, immediate intervention for stable mixed chimerism is not necessary for patients with non-malignant diseases.

This review summarizes current methods and clinical applications of chimerism analysis after allo-HSCT. 

## 2. Methods of Chimerism Analysis

### 2.1. Techniques of Chimerism Analysis

DNA differences between donor and recipient cells can be assessed by polymerase chain reaction (PCR). Analysis of DNA short tandem repeats (STR-PCR) has long been the predominant method of chimerism analysis. More recently, quantitative real-time PCR (qPCR) has been used to detect variations of single nucleotide polymorphisms (SNPs) or insertions/deletions (indels) with higher sensitivity than STR-PCR. Fluorescent in situ hybridization for sex chromatins (X/Y FISH) can be used for chimerism analysis in cases of sex-mismatched allo-HSCT. In addition, novel techniques such as digital PCR devices and next-generation sequencing (NGS) systems have emerged. Informativity and sensitivity for detecting recipient chimerism vary among methods (Table 1). Currently, most hematologic laboratories supporting allo-HSCT programs routinely use either STR-PCR or qPCR for chimerism analysis [7,8]. In the leukemic case with an available marker for minimal residual disease (MRD), qPCR, digital PCR, and NGS can also detect MRD along with recipient chimerism (Figure 1).

#### 2.1.1. STR-PCR

STRs are DNA sequential repeats of 2 to 6 base pairs that can distinguish one person’s genotype from another’s. The use of 12 or more STR markers distinguishes donor and recipient genotypes with 100% informativity. STR-PCR uses fluorescent-labeled primer pairs for donor-specific and recipient-specific short tandem repeats, followed by capillary electrophoresis, through which chimerism is estimated by the donor- and recipient-specific peaks of PCR products [9,10]. With 1 ng or more of DNA, STR-PCR has a 1–5% detection limit [2,11].

STR-PCR is the prevailing method of chimerism analysis used in >80% of hematology laboratories in allo-HSCT centers [7,8]. However, STR-PCR has some limitations, such as technical variability among laboratories and relatively low sensitivity, with detection limits that depend on the DNA [11,12,13,14,15,16]. Thus, it is important to implement technical standardization for STR-PCR [14,16].

#### 2.1.2. qPCR

In contrast to STR-PCR, qPCR is a faster and more sensitive method that can detect as low as approximately 0.1% recipient chimerism [11,17,18,19,20,21,22,23]. Recently, >20% of hematology laboratories have adopted qPCR for chimerism analysis [7,8]. A small amount of DNA is sufficient for qPCR, with sensitivity depending on the DNA sample size: ≤0.1% recipient chimerism is detectable in 100 ng DNA, whereas the detection limit of recipient chimerism is 0.1–1.0% with 10 ng DNA. The issues of qPCR are limited informativity, inconsistent quantitative accuracy, false positive results, and technical variations [2,11]. However, the use of 40 or more markers can achieve almost 100% informativity [17,24]. 

We recently conducted a study in Japan of qPCR kits (KMRtype/KMRtrack, GenDx, Utrecht, The Netherlands) labeled with the European Commission mark for in vitro diagnostics [17]. In that study, 39 indel markers (KMR markers) could distinguish all 65 Japanese donor/recipient pairs. In addition, the percentage of recipient chimerism measured by KMRtrack was well correlated with ratios of mixed DNA in virtual samples and with the percentage of chimerism in allo-HSCT recipients previously examined in clinical practice by STR-PCR [9,10,25] or in-house qPCR for SNPs [22]. KMRtrack showed better sensitivity with high specificity when compared to STR-PCR to detect recipient chimerism. In fact, an assessment of KMRtrack using virtual pairs consisting of DNA from 2 different individuals showed a good correlation between measured and simulated degrees of chimerism, with sensitivity high enough to detect thresholds of ≤0.3% from samples with as little as 10 ng of DNA [17].

#### 2.1.3. X/Y FISH

X/Y FISH targeting sex chromosome genes in blood cells or histology specimens can be used for chimerism analysis after sex-mismatched allo-HSCT [26,27]. Only about half of transplants are sex-mismatched, so X/Y FISH has limited clinical applicability. Thus, few laboratories are currently using X/Y FISH for chimerism analysis [8]. Recently, the RNA PrimeFlow™ system (Thermo Fisher Scientific, Waltham, MA, USA) has been used to detect *KDM5D* mRNA on the Y chromosome of lineage-specific cells, using RNA hybridization and flow cytometry. RNA PrimeFlow™ allows lineage-specific chimerism analysis without cell sorting with a 1% detection limit for sex-mismatched allo-HSCT [28]. Further validation is warranted.

#### 2.1.4. Digital PCR

Digital PCR techniques are available and even more sensitive than qPCR for chimerism detection [15,29,30,31,32]. Kliman et al. used droplet digital PCR (ddPCR) to achieve levels of detection and quantification of 0.008% and 0.023%, respectively, in mixtures of DNA from 2 individuals using a plate with 240 ng DNA with 8 replicates for each dilution. Results of chimerism analysis with ddPCR correlated well with those with STR-PCR in samples from 8 allo-HSCT recipients. Furthermore, in patients who received clinical trials of allo-HSCT along with virus-specific T (VST) cells, chimerism of the VST cells in the background of recipient and donor cells was detectable in 11 of 12 cases (92%) [33]. Fortschegger et al. reported that ddPCR can detect 0.1% recipient chimerism in DNA from 20,000 cells [34]. Likewise, a crystal digital PCR (cdPCR) platform with 3-color multiplexing capacity has been reported to be a promising technique for chimerism analysis. The results of cdPCR correlated well with those of qPCR with KMRtrack. The cdPCR detected 0.1% recipient chimerism [30]. In both ddPCR and cdPCR techniques, KMR markers are applicable to chimerism analysis as commercially available markers [30,33].

#### 2.1.5. NGS

Next-generation sequencing (NGS) can detect multiple differences in SNPs, indels, and STRs simultaneously between donor and recipient for chimerism analysis, with high sensitivity [2,15,30,35,36]. Moreover, MRD is concurrently evaluable. Pedini et al. [30] showed reliable chimerism analysis by NGS, with a 0.1% detection limit that strongly correlated with the results of qPCR and STR-PCR. While current NGS systems are expensive and labor intensive, NGS is becoming more popular in clinical laboratories. NGS-based chimerism analysis may emerge as a promising alternative to STR-PCR and qPCR in the future [8]. 

### 2.2. Detection Limits and Definitions of Chimerism

Complete donor chimerism is when chimerism analysis does not detect a recipient-derived cell in PB or BM samples after an allo-HSCT transplant. However, the sensitivity of chimerism analysis varies with different methodologies and sample sizes. Accordingly, the definition of mixed chimerism may vary among studies from thresholds of <1% to 20% of recipient chimerism. In other words, from 80% to >99% of donor chimerisms can be deemed either complete donor chimerism or mixed chimerism, depending on the study. This variability complicates the assessment of chimerism analysis as a diagnostic tool and therapeutic guide. 

The American Society for Transplantation and Cellular Therapy (ASTCT) recently published a guideline that defined 5% of recipient chimerism as the detection limit for complete donor chimerism in lymphoid and myeloid lineages [37]. This aligns with the fact that detection limits of 1% to 5% are common among hematology laboratories using STR-PCR. On the other hand, recipient chimerism is associated with relapse of hematologic malignancies even at levels <1% when using sensitive methods such as qPCR, digital PCR, and NGS [12,17,19,38,39]. Thus, detection limits of 1% or less may be warranted in clinical situations such as AML or acute lymphoblastic leukemia (ALL) that otherwise lack an MRD marker. In addition, combining chimerism analysis with MRD detection is a promising option to prevent or treat post-HSCT relapse of hematologic malignancies as early as possible, with interventions including DLI [39,40,41,42,43,44]. It should be noted that the presence of recipient chimerism is less specific for the prediction of disease relapse, and it does not necessarily indicate an active hematologic malignancy [45]. Moreover, MRD detection with a sensitive method may precede the emergence of recipient chimerism at a time point prior to clinical relapse [39,44].

### 2.3. Timing of Assay

The ASTCT guideline attempted to standardize the assessment of hematopoietic reconstitution after allo-HSCT, including timing for chimerism analysis, by consensus of expert adult and pediatric hematologists. Both adult and pediatric panels recommended routine examination by chimerism analysis at 1, 3, 6, and 12 months after allo-HSCT [37]. However, a subsequently published worldwide survey [8] showed that although over 90% of laboratories performed chimerism analysis at 1 month after allo-HSCT, only about 70% examined chimerism at 3 and 6 months, and only 60% at 1 year after allo-HSCT, suggesting that the timing and frequency of chimerism assays should be more standardized. On the other hand, chimerism analysis at 1 month or earlier after allo-HSCT has been reported to predict graft rejection [46,47,48,49,50].

### 2.4. Cell Types

Various hematopoietic cells, such as whole-blood and bulk BM cells, as well as lineage-specific cells—including T cells, myeloid cells, B cells, natural killer cells, and hematopoietic progenitor cells (HPCs)—are applicable to chimerism analysis (Table 2) [45,51,52]. Lineage-specific chimerism analysis may show higher sensitivity compared to whole-blood chimerism analysis [4,42,53]. Chimerism analysis performed on lineages of malignant cells has shown high sensitivity for detecting relapses of AML, myelodysplastic syndrome (MDS), and ALL [42]. In addition, it has been suggested that various lineages of hematologic and immunologic cells are reconstituted in different manners [13,25,54,55,56,57], which may lead to split chimerism, that is, different chimerisms among different lineages. T-cell depletion and the use of reduced-intensity conditionings (RICs) for allo-HSCT are often associated with mixed chimerism, especially in T cells [58,59,60,61,62]. Also, in allo-HSCT recipients who receive RIC, T cell chimerism may fluctuate more than B cell chimerism at different time points [53]. 

Although chimerism analyses performed on multiple lineages are informative, the most commonly used sample is whole blood, followed by T cells and myeloid cells; less than 20% of laboratories use HPCs, B cells, or natural killer cells in clinical care [8]. Other issues are that appropriate detection limits in lineage-specific chimerism are not well established, and data comparing whole-blood chimerism with lineage-specific chimerism are lacking. A combination of T cell and whole-blood chimerism assays up to day +90 in patients who received RIC may be of value to appropriately interpret chimerism kinetics [26]. 

#### 2.4.1. Whole-Blood and Bulk Bone Marrow Cells

PB whole-blood samples are most frequently used for chimerism analysis, with approximately 80% of laboratories doing so [8]. Whole-blood samples for chimerism analysis need less manipulation than those for lineage-specific analyses [7,8,63], thus making analysis easier to perform. In patients with leukocytopenia, including graft failure, whole-blood chimerism is used instead of lineage-specific chimerism when the number of lineage-specific cells is insufficient [66]. 

Chimerism of bulk BM cells may be more sensitive than PB whole-blood chimerism [51]. BM samples are also useful to predict or diagnose graft failure in patients with low PB leukocyte counts. BM chimerism assessed at day +14 was an independent factor predicting myeloid engraftment after CBT in adults with hematologic malignancies [46]. It has been proposed that CBT recipients without neutrophil recovery at day +21 after transplantation should be evaluated with BM chimerism at days +21 and +28, rather than PB chimerism, to inform the search for a backup graft in case engraftment does not ensue [50].

A large retrospective study of 688 patients with hematologic malignancies indicated that whole-blood chimerism was comparable to T cell chimerism at day +100 for predicting outcomes: whole-blood mixed chimerism (donor chimerism ≤ 90%) at day +100 was significantly associated with increased relapse, worsened progression-free survival, and worsened overall survival (OS) [58]. Secondary graft failure with mixed chimerism and graft rejection with complete recipient chimerism, evaluated by chimerism analysis with either whole-blood samples or bulk BM cells, was associated with poor outcomes after allo-HSCT for aplastic anemia [74].

#### 2.4.2. T Cells

Mixed chimerism is most often observed in T cells than in other cell types [67]. About 70% of laboratories use T cells for chimerism analysis in clinical settings [8]. In research settings, T cell chimerism has been extensively evaluated in a wide variety of studies [8,47,57,60,61,63,64,65,66,67,68,69]. CD3 is used as a standard marker for T cells, and CD3^+^CD4^+^ and CD3^+^CD8^+^ T cell chimerisms have also been evaluated in some studies [41,47,67,68]. 

Various studies have correlated mixed T cell chimerism with relapse of hematologic malignancies. For instance, a relatively large retrospective study indicated that decreasing donor T cell chimerism is significantly associated with shorter survival after allo-HSCT for AML and MDS [57]. Another large study indicated that mixed T cell chimerism (donor chimerism ≤ 85%) at days +90 to +120 in patients in apparent remission from AML and MDS was associated with increased 3-year disease progression, although mixed T cell chimerism was not associated with relapse in the cohort without remission [65]. 

T cell chimerism is also a surrogate marker of engraftment or rejection of donor cells [47,64]. T cell chimerism analysis is technically feasible even for evaluations earlier than 1 month (i.e., 2 to 4 weeks) after allo-HSCT, with less than 50% T cell chimerism indicating a risk of graft rejection [47]. 

#### 2.4.3. Myeloid Cells

Myeloid chimerism may best inform the status of active, donor-derived hematopoiesis, rather than T cell chimerism [70]. Myeloid mixed chimerism can also indicate a relapse of myeloid malignancies. Lindahl et al. recently reported that complete donor myeloid chimerism (>99.8% in CD33^+^ cells), evaluated by STR-PCR or qPCR, was significantly correlated with a lower rate of relapse of AML [72]. CD33, as well as CD14, CD15, and CD66b, are used as markers for myeloid chimerism analysis [13,56,63,65,70,71].

#### 2.4.4. HPCs

Decreasing CD34^+^ HPC chimerism is an independent risk factor for relapse of AML and MDS. The sensitivity of CD34^+^ chimerism for patients with CD34^+^ AML and MDS was comparable to the detection of leukemia-specific genetic abnormalities with PCR-based methods [4]. Even in patients with stable donor chimerism in PB and BM before relapse, decreasing CD34^+^ HPC chimerism is detectable and associated with subsequent relapse, suggesting the value of CD34^+^ HPC-specific chimerism analysis to detect residual neoplastic cells after allo-HSCT for AML or MDS [73]. It was also reported that the appearance of recipient CD34^+^ cells, as well as CD8^+^ cells, shows a significant association with relapse of pediatric ALL [68].

## 3. Chimerism Analysis Guiding Management of Allo-HSCT Recipients

Complete donor chimerism generally correlates with better outcomes, whereas mixed chimerism, in cases of hematologic malignancies or post-transplant cytopenia, suggests relapse or graft failure, respectively. Possible interventions for mixed chimerism include adjusting the immunosuppression regimen, performing DLI, boosting CD34^+^ HPCs, and repeating allo-HSCT. However, the management of mixed chimerism is still a matter of debate. Also, the recent ASTCT guideline indicated that the word “recovery” is more appropriate than “engraftment” because confirmation of donor-sourced engraftment requires proof of some donor chimerism [37]. 

### 3.1. Impaired Hematopoietic Recovery without Relapse

Neutrophil recovery that fulfills engraftment criteria is defined as the first of 3 consecutive days with an absolute neutrophil count ≥0.5 × 10^9^/L (Table 2) [62]. Not achieving this absolute neutrophil count by day +28 in BMT and PBSCT or day +42 in CBT is used to define primary graft failure. In addition, sustained platelet counts >20 × 10^9^/L and hemoglobin concentrations >80 g/L without transfusion are criteria for the formal definition of engraftment. Chimerism analysis can guide diagnosis in cases of cytopenias with suspected graft failure. Increasing recipient chimerism is likely to indicate graft rejection caused by immune rejection of donor cells mediated by recipient cells. Poor graft function is characterized by cytopenias that require cellular blood component transfusions and/or growth factor support in the absence of alternative explanations, such as disease relapse, drugs, or infection [62,75]. Either graft rejection or poor graft function can lead to graft failure. Chimerism analysis is especially recommended to confirm donor-derived hematopoiesis in patients who received RIC, as recipient chimerism could be dominant [62]. Secondary graft failure shows cytopenias after initial engraftment not related to relapse, infection, or drug toxicity. An assessment of secondary graft failure should include complete blood counts, BM cellularity, and chimerism analysis. The ASTCT guideline includes the word “recovery” instead of “engraftment” to reflect the fact that engraftment requires proof of at least partial donor chimerism, although both adult and pediatric physician panels in the ASTCT endorsed prior definitions (Table 3) [37]. 

**Figure 2:**
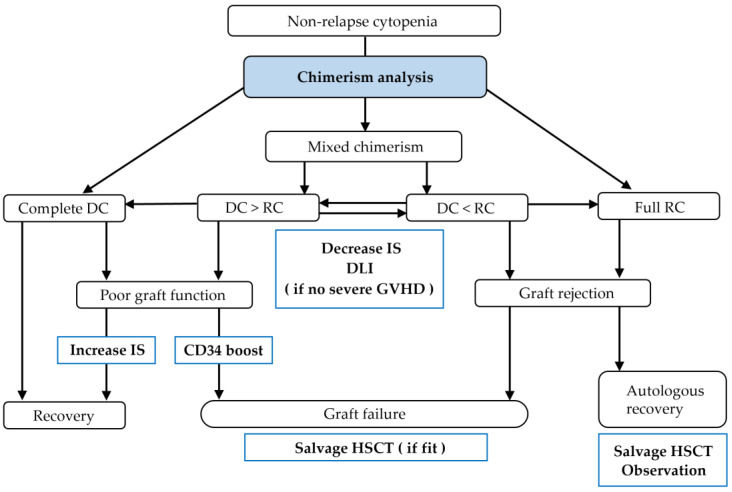
Interventions for non-relapse cytopenias after allo-HSCT. DC, donor chimerism; RC, recipient chimerism; GVHD, graft-versus-host disease; IS, immunosuppressors.

Chimerism analysis is crucial for determining treatment strategies for patients with non-relapsing cytopenias after allo-HSCT (Figure 2). In such cases, no or minimal donor chimerism may be associated with graft rejection, whereas complete donor chimerism may mean poor graft function. In addition, there are cytopenic patients with mixed chimerism who may develop bone marrow failure, graft rejection, or resolution of their cytopenia [75,76]. Risk factors for graft failure include the use of RIC and/or CBT, the presence of HLA antibodies, low infused cell number, viral infection, and various other causes [62]. A scoring system of risk for primary graft failure at day +21 after BMT and PBSCT for hematologic malignancies has been developed: age (<30, 1 point), Karnofsky score (<90%, 1 point), disease [AML/ALL, 0; MDS, 1; chronic lymphocytic leukemia or chronic myeloid leukemia (CML), 2; and myeloproliferative neoplasms, 3 points], disease status (advanced AML/ALL/CML, 1 point), HLA match and relativity (mismatched unrelated, 2 points), graft type and total nucleated cells (BM cells ≤ 2.4 × 10^8^/kg, 1 point; PBSCs, 2 points), conditioning regimen (no TBI, 2 points), and GVHD prophylaxis (no calcineurin inhibitor + methotrexate, 2 points; T-cell depletion, 3 points). A score ≥6 at day +21 had a positive predictive value of 28% to 36% for graft failure [62,77]. Thus, in patients with a high risk of primary graft failure, chimerism analysis early after allo-HSCT is particularly important.

The main treatment for graft failure with graft rejection is a salvage second allo-HSCT. Most studies involving salvage second allo-HSCT for graft failure are retrospective and small with heterogeneity in conditioning regimens and graft selections as well as timing of onset; these studies presumably include primary and secondary graft failure. In general, OS after the second allo-HSCT for graft failure is poor due to high rates of treatment-related mortality associated with infections [76]. However, recent studies have shown acceptable OS after salvage second allo-HSCT, including CBT or allo-HSCT from haploidentical related donors [78,79,80,81,82,83]. No treatments other than salvage second allo-HSCT are established for graft failure with graft rejection, although some investigational drugs, such as an interferon-gamma inhibitor, have been tried [76]. Interventions for graft failure without graft rejection are more variable than those with graft rejection and are based on specific situations [3,37,75,76]. Cytopenias in the presence of dominant or complete donor chimerism associated with poor graft function, including donor-type aplasia, are well known [76,84]. The pathophysiology of poor graft function remains largely unknown, and proposed mechanisms include functional impairment and exhaustion of donor hematopoietic stem cells, alloreactivity of donor T cells to microenvironments of hematopoiesis, inflammatory cytokines harmful to hematopoietic stem cells and microenvironments, GVHD, and viral infection. For graft failure without graft rejection, an increase in or withdrawal of immunosuppressors, salvage second allo-HSCT, stem cell boosts with CD34^+^ cells, mesenchymal stem cells, and thrombopoietin receptor agonists have been attempted, but evidence supporting these interventions is limited [75,76,85,86,87,88,89,90,91]. Recently, Shahzad and colleagues conducted a meta-analysis for stem cell boosts with CD34^+^ cells for poor graft function [92]. There were 209 patients in seven matched studies of poor graft function [85,86,87,88,89,90,91] available for this meta-analysis. The median time from allo-HSCT to stem cell boosts was 138 days (range, 113 to 450 days). OS ranged from 80% at 1 year to 40% at 9 years. Rates of non-relapse mortality and death due to relapse were 27% (95% CI, 17% to 40%) and 17% (95% CI, 11% to 23%), respectively, indicating an acceptable outcome of stem cell boosts with CD34^+^ cells for post-allo-HSCT poor graft function. A limitation was that the literature used in this meta-analysis did not include high-quality randomized evidence. 

### 3.2. Mixed Chimerism in Non-Malignant Diseases

Patients receiving allo-HSCT for non-malignant diseases frequently develop mixed chimerism, and the requirements for intervention depend on the specific underlying disease, condition, GVHD prophylaxis, and cytopenia [37,74,82,93,94,95,96,97]. Donor-derived cells with a stable mixed chimerism can sufficiently improve hematopoiesis, restore immunocompetence, or lead to the production of the deficient enzyme, basically without risk of relapse of malignant disease, in patients with non-malignant diseases (Figure 1b) [3,26]. Indeed, stable mixed chimerism without signs of graft rejection did not influence survival rates in allo-HSCT recipients with non-malignant diseases [53,93,95]. By contrast, either a decrease in donor chimerism or poor graft function accompanied by cytopenias may result in graft failure [3,74,94,96]. The recommended donor chimerism levels vary according to disease and lineage specificity. For instance, severe combined immunodeficiency requires almost complete donor chimerism in T, B, and natural killer cells, whereas >50% donor chimerism in myeloid cells suffices in chronic granulomatous disease [98]. In aplastic anemia, 18 patients with stable mixed chimerism having <15% recipient chimerism did not progress to graft rejection, whereas donor cells were eventually rejected in most patients with progressively increasing recipient chimerism, even at levels <10% [99], suggesting that ≥85% stable donor chimerism can maintain sufficient hematopoiesis after allo-HSCT for aplastic anemia without further intervention.

Kako and colleagues reported an association between mixed chimerism and secondary graft failure in adult patients with aplastic anemia [74]. They divided recipients of BMT or PBSCT from a Japanese registry database into four groups: stable mixed chimerism (group 1, n = 26), stable mixed chimerism requiring some cytokine and/or transfusion support (group 2, n = 16), secondary graft failure with mixed or no donor chimerism, representing mostly graft rejection (group 3, n = 19), and secondary graft failure with complete donor chimerism, representing poor graft function (group 4, n = 17). OS was significantly inferior in group 3 (1 year, 52.1%; 5 years, 52.1%) and group 4 (1 year, 82.4%; 5 years, 56.3%), but not in groups 1 and 2, compared to the controls without mixed chimerism or graft failure (n = 340; 1 year, 90.4%; 5 years, 83.5%). In group 3, 12 of 19 recipients received a salvage second allo-HSCT, of which 10 cases were rescued. Among 17 cases in group 4, immunosuppressors were increased in 7 patients, and 8 patients underwent salvage second allo-HSCT. The cytopenias were ameliorated by the increase in immunosuppressors in five of seven patients, while second allo-HSCT was successful in four of eight patients. In contrast, other studies have shown that mixed chimerism can be stabilized or even converted to complete donor chimerism by DLI in patients with non-malignant diseases. However, a majority of patients do not show sufficient benefit from increased donor chimerism, and there is the potential risk of DLI to induce GVHD, which should be assiduously avoided in patients with non-malignant diseases [3,94,100]. 

In brief, interventions for cytopenias after allo-HSCT for non-malignant diseases include an increase in or the withdrawal of immunosuppressors, DLI, and salvage second allo-HSCT; treatment strategies should take account of donor chimerism and the overall status of the patient.

### 3.3. Mixed Chimerism in Hematologic Malignancies

Chimerism analysis allows the assessment of persisting or reappearing recipient cells. Mixed chimerism may reflect the survival of malignant cells, the survival of non-malignant recipient hematopoietic cells, or a combination of both in hematologic malignancies (Figure 1a). 

Immunotherapeutic interventions, including the withdrawal or tapering of immunosuppressors and DLI, can convert mixed chimerism to complete donor chimerism and restore the graft-versus-leukemia effect, which may prevent hematological relapse [3]. It is not always suitable to use chimerism analysis as an indicator of relapse; STR-PCR sensitivity is low, with a detection limit of 1–5%, possibly including a mixture of malignant and non-malignant hematopoietic cells. Chimerism analysis for lineage-specific cells compatible with leukemic cells (e.g., CD34), the use of more sensitive methods, and serial analyses may be helpful in predicting risks for relapse.

Studies evaluating both recipient chimerism and MRD in acute leukemia after allo-HSCT have shown that MRD detection preceded the emergence of >1% recipient chimerism measured by STR-PCR; however, increasing recipient chimerism was associated with relapse [41,44]. A study using qPCR, which is more sensitive than STR-PCR, demonstrated a relapse rate of 55% at 2 years in acute leukemia patients with >1.0% recipient chimerism, whereas no acute leukemia patients with < 1.0% recipient chimerism showed relapse [6]. The KIM-PB study, which prospectively investigated the relapse of AML in correlation with sensitive chimerism analysis using qPCR with the KMR kits for recipients of allo-HSCT, revealed that increasing recipient chimerism could predict relapse even at low levels: 0.13% and 0.24% for whole blood and BM, respectively [38]. These studies suggested that increasing recipient chimerism may predict relapse, even at a low level.

Post-allo-HSCT relapse rates are still high in hematologic malignancies [1]. However, relapse treatment options and their respective efficacies are extremely limited. Therefore, preemptive or prophylactic strategies to prevent overt relapse are crucial. Currently, preemptive or prophylactic DLI and maintenance medication, e.g., azacytidine and FLT3 inhibitors, are available options for the prevention of overt relapse [4]. The detection of MRD and recipient chimerism should guide these preemptive strategies, although suitable MRD markers for monitoring the relapse of hematologic malignancies are limited [3,4]. In this respect, chimerism analysis can almost always distinguish donor/recipient pairs and identify the proportion of recipient-derived cells, but the overall proportion may contain both malignant and non-malignant cells.

**Figure 3:**
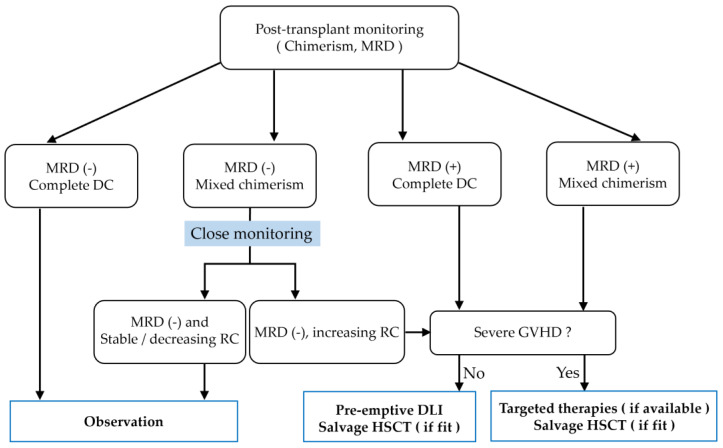
Interventions based on chimerism analysis after allo-HSCT for leukemia in the presence of residual recipient chimerism and/or minimal residual disease (MRD) with a potential to relapse into malignancy. Targeted therapies include tyrosine kinase inhibitors, monoclonal antibodies, and cellular therapies. DC, donor chimerism; RC, recipient chimerism; GVHD, graft-versus-host disease; IS, immunosuppressors; DLI, donor lymphocyte infusion.

It has been strongly suggested that prophylactic or preemptive DLI for patients with MRD or mixed chimerism is more beneficial than therapeutic DLI after overt relapse [5,45,101,102]. Preemptive DLI can eliminate MRD with the conversion of mixed chimerism to complete donor chimerism, increasing the likelihood of survival [6,41,103,104,105]. Rettig and colleagues have reported a correlation between the timing of preemptive DLI and outcome: the OS of patients who received DLI when they had only mixed chimerism was superior to the OS of patients who received DLI after the detection of a genetic MRD marker with no sign of hematologic relapse [101]. Together, these findings indicate that early warning at a time when the patient has a low tumor burden may contribute to increased efficacy of the graft-versus-leukemia effect by preemptive DLI (Figure 3).

## 4. Discussion

Chimerism analysis serves to characterize graft failure, graft rejection, poor graft function, and relapse, as well as engraftment or hematopoietic recovery. In allo-HSCT, donor age, HLA match, donor types and stem cell sources, the use of T-cell depletion for GVHD prophylaxis, and the intensity of conditioning regimens all can affect chimerism and engraftment status. The importance of chimerism analysis continues to grow with advances in transplant practice that increase the volume and variety of allo-HSCT procedures: more donors, including HLA-haploidentical relatives; improvements in safety and tolerability; and the use of RIC to accommodate more patients. Requirements of donor chimerisms to overcome non-malignant diseases or prevent the relapse of malignant diseases are widely variable. RIC is preferentially used for patients with comorbidities as well as those with disorders that are controllable with mixed chimerism, although the stability of donor chimerism should be carefully monitored.

How should mixed chimerism guide patient care? Stable mixed chimerism is fully acceptable for some non-malignant diseases, but decreasing chimerism prompts tapering or the withdrawal of immunosuppressors. There is an opinion that DLI is generally not of benefit, since it can induce unnecessary GVHD, which should be avoided in non-malignant diseases [98]. However, in real-world settings, DLI remains an option in non-malignant cases with down-trending CD3 and/or CD33 donor chimerism, according to ASTCT pediatric and adult expert panels [37]. Alternatively, an early rapid drop in donor chimerism often requires a second allo-HSCT, whereas a gradual decline can be salvaged by an adjustment of immunosuppressors in non-malignant diseases [74,98]. On the other hand, mixed chimerism in patients with high-risk hematologic malignancies usually needs intervention, including prophylactic or preemptive DLI, to prevent relapse [3,45]. 

In non-malignant disease cases, mixed chimerism is particularly an issue when donor chimerism is rapidly decreasing. On the other hand, residual recipient chimerism can be the source of relapse in hematologic malignancies. Therefore, the degree of sensitivity needed depends on underlying conditions, including the presence or absence of malignancy. For patients with non-malignant diseases, current standard STR-PCR techniques with detection limits of around 5% suffice for patient management, whereas highly sensitive chimerism analysis may help to predict relapse prior to other clinical or hematological evidence. Lineage-specific chimerism analysis, more sensitive techniques like qPCR or digital PCR for chimerism analysis, and combinations of chimerism analysis with MRD assays are reasonable strategies to detect minimal malignant recipient cells for intervention to prevent hematological relapse. NGS is a promising method because it can simultaneously detect MRD and recipient chimerism [7]. Considering that NGS is becoming more available in clinical laboratories, NGS-based chimerism analysis might supplement or displace STR-PCR and qPCR in the future.

## 5. Conclusions

Chimerism analysis plays a crucial role in monitoring patients after allo-HSCT to ensure engraftment or find evidence of relapse. STR-PCR is still the gold standard for chimerism analysis, while more sensitive methods are emerging, such as qPCR and NGS, which can also detect MRD in malignancies. Thus, sensitive chimerism analysis—along with MRD testing—may pave the way for better outcomes in allo-HSCT by guiding clinical care. However, best practices are yet to be established, in part because technological evolution continues to improve the sensitivity and informativity of assays, and chimerism-guided interventions continue to be tested. Standardization of patient care in the future would benefit from initiatives to standardize data collection and analysis now, institutionally, nationally, and internationally.

## Figures and Tables

**Table 1:** Methods of chimerism analysis.

Technique	Applications	Markers	Sensitivity *	Informativity
STR-PCR	Chimerism	STRs	1–5%	=100%
qPCR	Chimerism, MRD	SNPs, indels	=0.1%	90–100%
X/Y FISH	Chimerism after sex-mismatched transplantation	X/Y chromosome	≤5%	≒50%
Digital PCR	Chimerism, MRD	SNPs, indels	0.01–0.1%	90–100%
NGS	Chimerism, MRD	SNPs, indels	0.01–1%	100%

* Detection limits depend on the DNA sample quantity and quality. STR, short tandem repeat; qPCR, quantitative PCR; X/Y FISH, fluorescence in situ hybridization for sex chromatins; NGS, next-generation sequencing; MRD, minimal residual disease.

**Table 2:** Major cell types used for chimerism analysis.

Cell Type	Markers	Property	Applications	Advantages	Disadvantages	References
Whole blood	–	PB	Routine use Engraftment confirmation Diagnosis of graft failure	Easy to obtain sample Less manipulation	Low sensitivity Low specificity	[7,8,63]
Bulk marrow	–	BM	Engraftment confirmation Diagnosis and prediction of graft failure	High sensitivity Useful for leukopenic patients	Low specificity	[46,51]
T cells	CD3 CD4, CD8	PB	Surrogate for graft rejection or relapse	High frequency of MC Widely available data	Indirect for hematopoietic reconstitution	[8,47,57,60,61,63,64,65,66,67,68,69]
Myeloid cells	CD33 CD14, CD15, CD66b	BM PB	Surrogate for relapse of AML, MDS	Best information of hematopoietic origin	Limited available data	[13,56,63,65,70,71,72]
HPCs	CD34	BM	Surrogate for relapse of AML, MDS, ALL	High sensitivity for predicting relapse	Difficulty in obtaining sample	[68,73]

HPCs, hematopoietic progenitor cells; PB, peripheral blood; BM, bone marrow; MC, mixed chimerism; ALL, acute lymphoblastic leukemia.

**Table 3:** Definitions of hematopoietic recovery after allo-HSCT.

	Definition	Major Chimerism
Engraftment	First of 3 consecutive days with ANC ≥ 0.5 × 10^9^/L, PLT > 20 × 10^9^/L, Hb > 80 g/L (free of transfusion requirement)	Donor
Primary graft faliure	ANC < 0.5 × 10^9^/L by day +28 in BMT and PBSCT (by day +42 in CBT)	Depends on cause -Poor graft function: donor -Graft rejection: recipient
Secondary graft faliure	ANC < 0.5 × 10^9^/L after initial engrafment not related to relapse, temporal infection, or drug toxicity	
Poor graft function	Two or three cytopenias >2 weeks after day +28	Donor
Graft rejection	Immune rejection of donor cells	Recipient

ANC, absolute neutrophil count; PLT, platelet count; Hb, hemoglobin concentration; CBT, cord blood transplantation.

## Data Availability

No new data were created or analyzed in this study. Data sharing is not applicable to this article.
